# Effect of Stocking Density and Dietary Protein Level in Biofloc System on the Growth, Digestive and Antioxidant Enzyme Activities, Health, and Resistance to Acute Crowding Stress in Juvenile Common Carp (*Cyprinus carpio*)

**DOI:** 10.1155/2022/9344478

**Published:** 2022-09-02

**Authors:** Hossein Adineh, Mahdi Naderi, Hojatallah Jafaryan, Mohammad Khademi Hamidi, Morteza Yousefi, Ehsan Ahmadifar

**Affiliations:** ^1^Department of Fisheries, Faculty of Agriculture and Natural Resources, Gonbad Kavous University, Gonbad Kavous, Golestan, Iran; ^2^Department of Fisheries, Iran Fisheries Organization, Behbahan, Khouzestan, Iran; ^3^Department of Veterinary Medicine, Peoples' Friendship University of Russia (RUDN University), Moscow, Russia; ^4^Department of Fisheries, Faculty of Natural Resources, University of Zabol, Zabol, Iran

## Abstract

This study is aimed at evaluating the effects of stocking densities and dietary protein levels in the biofloc system on the performance of common carp. Fish (12.09 ± 0.99 g) were transferred to 15 tanks: fish reared at 10 kg/m^3^ as medium density and fed 35% (MD35) or 25% (MD25) protein and fish reared at 20 kg/m^3^ as high density and fed 35% (HD35) or 25% (HD25) protein in the biofloc system and control fish reared at MD and fed 35% protein in clear water. After 60 days, fish were subjected to crowding stress (80 kg/m^3^) for 24 h. The growth of fish was highest in MD35. The feed conversion ratio was lower in MD35 compared to the control and HD groups. The amylase, lipase, protease, and superoxide dismutase and glutathione peroxidase activities in the biofloc groups were significantly higher than in the control. After crowding stress, cortisol and glucose levels in biofloc treatments were significantly decreased compared to the control. After 12 and 24 h stress, lysozyme activity in MD35 was significantly lower than in the HD treatments. Overall, the biofloc system with MD could improve growth and robustness against acute stress in fish. Also, biofloc could compensate 10% reduction of protein in common carp juvenile diet when reared in MD.

## 1. Introduction

World aquaculture production of cultivated aquatic animals grew on average by 5.3% in the duration 2001–2018 [1]. To raise aquaculture productivity and profitability, novel and high-density culture methods are usually desirable and used [2]. However, high stocking density combined with higher protein content in diet in intensive aquaculture produces large quantities of waste products (nitrogen and phosphorus), which negatively affect the natural aquatic ecosystems. Thus, suitable methods are needed to preserve the environment and enhance sustainable aquaculture [3, 4]. Also, the development of aquaculture production is limited due to the pressure on the environment by its reliance on fish meal. Aquaculture using biofloc technology combines the removal of waste products from the water with the production of microbial flocs, which can *in situ* be consumed by the fish as proteinaceous food [4, 5]. The addition of a carbon source (carbohydrates) to the biofloc culture system stimulates the development of heterotrophic bacteria and nitrogen uptake which produced microbial proteins. These microbial flocs can be consumed by the fish resulting in continuous recycling nutrients and reuse of food in the biofloc system [6, 7]. Biofloc technology reduces water exchange in culture systems through enhancing water quality and simultaneously provides low cost biofloc (rich in protein), which can use as an additional food in aquaculture [4, 5].

Fish farmers try to raise productivity and profitability by increasing the fish stocking density, despite its unfavorable effects on farmed fish [8, 9]. In the biofloc conditions, the fish culture density could be higher than that in the clear water tanks [9, 10]. So, the biofloc system had anticrowding stress efficacy [8, 9]. Adineh et al. [8] indicated that the high stocking density reduced the growth and negatively influenced the welfare of common carp in clear water tanks, but biofloc improved water quality, growth and feed conversion ratio, immune function, and robustness against acute crowding stress in the intensive culture. Bakhshi, Najdegerami, Manaffar, Tukmechi, and Farah [11] exhibited that biofloc enhanced water quality and growth performance of common carp. Data on the stocking density of common carp using the biofloc system remains scarce. Adineh et al. [8] recommended an initial stocking density of 12 kg/m^3^ for the intensive cultivation of this species in biofloc conditions. Optimal density plays an important role in raising profit of fish production; thus, investigation is needed to determine the most suitable rearing density for better performance in the biofloc conditions. Moreover, it was reported that the crowded (chronically stressed) common carp was more susceptible to an additional acute confinement stress [12]. Acute stress challenge has been applied to assess the effects of chronic stress [13]. So, further studies are needed to investigate whether higher culture densities (chronic stress) influence the capacity of fish to combat an additional acute crowding stress, thus being more susceptible to acute stress.

Feed accounts for further than 50% of operational costs in fish production. In the biofloc system, the level of protein in the diet can be decreased and microbial flocs can be consumed as a supplemental protein source [3, 14]. Interestingly, the growth of Nile tilapia (*Oreochromis niloticus*) fed 20% dietary protein and cultured in the biofloc system with wheat milling by-product significantly surpassed those fed 30% dietary protein and cultured in a clear water system [14]. According to Nguyen, Trinh, Baruah, Lundh, and Kiessling [15], growth, feed intake, and protein efficiency were higher in juvenile Nile tilapia reared in the biofloc-RAS (recirculating aquaculture system) than fish kept in the clear water-RAS system. The feed conversion ratio was influenced by the protein level and by the availability of biofloc, with a general lower value in the biofloc-RAS and fish fed higher protein containing diets [15]. Furthermore, Yu, Huang, Du, Li, and Wu [16] showed that despite the low protein level in diet, biofloc has the potential to enhance growth and mitigate copper-induced immunosuppression and oxidative stress in juvenile *Rhynchocypris lagowski*. So, preparing low-protein diet decreases production costs and the influence on the environment by decreasing nitrogen input and fish meal usage in the diet ([17]; W. J. [18]). However, there was little study to investigate the performance of common carp cultured in a biofloc system with respect to different protein levels in diet. Ebrahimi, Akrami, Najdegerami, Ghiasvand, and Koohsari [19] reported that biofloc could not compensate 15% protein deficiency (drop from 35% to 20%) in common carp juvenile diet, but using rice bran+sugarcane molasses in the biofloc system enhances growth and immune and antioxidant responses of fish when fed 30% dietary protein. Thus, the lower protein level should reduce feed cost without negative effect on the growth and performance of common carp.

The determination of optimal levels of stocking density and dietary protein can enhance feed efficiency and fish growth and contribute to access the best usage of the biofloc culture system. So, the present study was performed to investigate the effects of stocking density and reducing the dietary protein level in a biofloc system on growth, digestive activity, antioxidant and innate immune parameters, and acute crowding stress response in common carp juveniles. The results of this experiment can be very practical to fish farmers.

## 2. Materials and Methods

### 2.1. Fish and Experimental Conditions

The experiment was performed at the fisheries laboratory, Faculty of Agriculture and Natural Resources, Gonbad Kavous University (Golestan, Iran), in compliance with the ethics and University guidelines for the care and use of animals. Common carp (*Cyprinus carpio*) fingerlings were obtained from a local fish farm (Sari, Mazandaran province, Iran). The fish were kept in two fiberglass tanks (water volume, 1000 L) and fed a diet containing 35% crude protein (Table 1) three times daily at 3% of the total fish biomass for two weeks to allow them to acclimate to laboratory conditions. Then, 1050 healthy common carp (12.09 ± 0.99 g mean body weight) were randomly transferred to 15 cylindrical fiberglass tanks (water volume, 60 L) at two initial stocking densities of 10 kg/m^3^ as medium stocking density (MD, 50 fish per tank) and 20 kg/m^3^ as high stocking density (HD, 100 fish per tank). The trial was designed in a 2 × 2 factorial design with two stocking densities (10 and 20 kg/m^3^) and two dietary crude protein levels (35 and 25%) in a biofloc system. Overall, four biofloc and one clear water (control) treatments (with three replicates of each one) were established: fish reared at medium density and fed diets containing 35% (MD35) or 25% (MD25) crude protein and fish reared at high density and fed diets containing 35% (HD35) or 25% (HD25) crude protein in the biofloc system and control fish (reared at medium density and fed a diet containing 35% crude protein in clear water system).

Ten days before the start of the experiment, microbial flocs stock (inoculum) tanks were initiated as described by Adineh et al. [8]. Briefly, 200 L of the water from fish acclimatization tanks was transferred to two cylindrical tanks, and the total ammonia nitrogen (TAN) level was determined. Beet molasses as a carbon source was added to the tanks daily at a carbon : nitrogen (C : N) ratio of 15 : 1 according to Crab et al. [4]. Tank aeration was ceased when the TAN level declined to almost zero and total suspended materials raised to 300 mg/L; then, the experimental tanks were inoculated with 200 mL/L of microbial flocs on the first day of the experiment [20].

Fish were hand-fed the experimental diets three times a day (08:30, 12:00, and 15:30 h) for 60 days at 2.5% of the total fish biomass in each tank, regulating the daily amount of food every two weeks based on the fish biomass. In the clear water (control) group, no carbon source was added to the tanks, and approximately 40% of the water was replaced every two days. In the biofloc tanks, freshwater was only added to compensate water loss caused by evaporation. Moreover, the biofloc tanks were supplied daily with carbon source (beet molasses) to adjust the C : N ratio of 15 : 1 for optimum production of biofloc according to Crab et al. [4]. Our previous study showed that this C : N ratio was appropriate for the common carp [8]. The light condition was adjusted at 12:12 h light/dark, and tanks were supported by air stones to provide suitable dissolved oxygen and robust water agitation using an air pump.

### 2.2. Experimental Diets

Two diets with 35 and 25% crude protein levels were formulated for common carp fingerlings (Table 1). All ingredients were thoroughly mixed and pelleted using an electronic meat grinder producing food pellets with diameter 3 mm. All diets were air-dried and then stored at 4°C until use.

### 2.3. Chemical Composition of Diets and Biofloc

At the end of the trial, the biofloc produced in biofloc tanks were collected, settled, concentrated, dried in an oven at 105°C, and then subjected to a chemical composition analysis. The proximate composition of the experimental diets and biofloc samples was analyzed in triplicates according to the standard methods [21].

### 2.4. Water Quality Monitoring

Water temperature, salinity, dissolved oxygen, electrical conductivity (EC), and pH were measured daily in the tanks using a portable multimeter (Hach HQ40d, Loveland, Colorado, USA). Total dissolved solids (TDS) were analyzed daily (hand-held meter, HANNA® instrument, India). Total ammonia nitrogen (TAN), nitrite, and nitrate levels were calorimetrically determined every 10 days following standard methods [22]. Total suspended solids (TSS) were measured every 10 days according to the method of Azim and Little [23]. Biofloc volume (BFV) was also measured using Imhoff cones every 10 days [3].

### 2.5. Growth Performance and Sampling

Weights of all fish from each tank were determined at the end of the 60-day trial after 24 h of starvation. Then, growth and feed utilization parameters were calculated. Also, 4 fish from each tank (12 fish per treatment) were randomly captured and anaesthetized with 200 mg/L clove powder [24], and blood samples were drawn from the caudal vein using sterile syringes. The time from capture to blood collection was <3 min to avoid cortisol increase due to handling during sampling. After coagulating, the serum samples were separated by centrifugation at 5000 × g for 10 min (Hermle Z36HK, Wehingen, Germany) and then stored at −80°C for later stress and immunological assays. After being bled, the digestive tracts and livers were also obtained from each fish for the measurement of digestive and antioxidant enzyme activities, respectively. After homogenizing in 100 mM Tris-HCl buffer with 0.1 mM EDTA and 0.1% Triton X-100 (pH 7.8) at a proportion of 1 g sample in 9 mL of buffer by an electric homogenizer (WIGGEN, D500, Berlin, Germany), the enzyme extracts were centrifuged at 25000 × g for 20 min at 4°C (Hermle Z36HK, Wehingen, Germany) and then supernatants obtained and stored at −80°C until enzymatic analysis [20].

### 2.6. Acute Crowding Stress Test

After 60 days of rearing and sampling, all fish from control and biofloc tanks were subjected to crowding stress (high density 80 kg/m^3^) for 24 h by decreasing the water level in tanks in a similar method to Adineh et al. [8]. Fish were not fed during the crowding stress and sampling because glucose and cortisol levels may be affected by feeding. There was no human interference during this time, and the tanks were kept in a quiet area. The tanks were aerated to maintain suitable dissolved oxygen level (>5 mg/L) and robust water agitation. After 6, 12, and 24 h of stress induction, the blood was randomly obtained from 4 fish in each tank (12 fish per treatment). The serum samples were separated as mentioned above and kept at −80°C until further analysis.

### 2.7. Digestive and Antioxidant Enzymatic Activities

The activity of amylase was determined using starch as the substrate according to the method developed by Langlois, Corring, and Fevrier [25]. The activity of lipase was determined using p-nitrophenyl myristate as the substrate [26]. The protease activity was assayed using casein as the substrate [27]. Liver catalase activity was assayed based on the method of Aebi [28]. The activity of liver superoxide dismutase (SOD) was also measured following the method of McCord and Fridovich [29]. Liver glutathione peroxidase (GPx) activity was determined using the method described by Noguchi, Cantor, and Scott [30]. The liver total antioxidant capacity (TAOC) level was measured using a commercial kit (ZEIIBio, Veltlinerweg, Germany) according to the manufacturer's guidance. The liver malondialdehyde (MDA) value was assayed following the thiobarbituric acid procedure [31].

### 2.8. Stress and Innate Immune Responses

The levels of serum cortisol were measured by the competitive enzyme-linked immunosorbent assay (ELISA) procedure using a commercial test kit (Monobind, Lake Forest, USA). Serum glucose content was determined based on the glucose oxidase procedure using a test kit (Pars Azmun, Karaj, Iran). The level of serum lactate was also determined using a commercial test kit (Biorex Diagnostics, Antrim, UK) according to the manufacturer's instructions. Serum lysozyme activity was measured by the turbidimetric analysis [32], based on the lysis of the lysozyme sensitive bacterium (*Micrococcus luteus*). Serum alanine aminotransferase (ALT) and aspartate transaminase (AST) activities were measured using assay kits (Pars Azmun, Karaj, Iran).

### 2.9. Data Analysis and Calculations

Statistical analysis was done using SPSS software version 24.0, and *p* < 0.05 was the agreed significance level. All the results were presented as means ± standard deviation(SD). Before analysis, the Shapiro-Wilk and Levene's tests were performed to confirm the normality and homogeneity of variance of the data, respectively. Then, the data were analyzed by one-way analysis of variance (ANOVA) to determine the significant differences between groups, followed by Duncan's multiple range tests to compare the means between groups. Two-way ANOVA was applied to check the effects of stocking density, dietary protein level, and their interactions excluding the clear water (control) group. Significant differences between the means achieved before and after crowding stress were analyzed by one-way ANOVA followed by Dunnett's test [24]. Dunnett *t*-tests treat one group as a control (before stress) and compare other groups (6, 12, and 24 h after stress) against it. The following parameters were also calculated:

(1) $$\begin{array}{c} \text{Weight gain}\left(\%\right)=100\times \frac{\text{final weight}–\text{initial weight}}{\text{initial weight}},\\ \text{Specific growth rate}\left(\text{SGR},\%{\text{day}}^{-1}\right)=100\times \frac{\ln\text{ final weight}–\ln\text{ initial weight}}{\text{days}},\\ \text{Feed conversion ratio}\left(\text{FCR},\text{g }{\text{g}}^{-1}\right)=\frac{\text{dry feed intake}}{\text{final weight}–\text{initial weight}},\\ \text{Protein efficiency ratio}\left(\text{PER},\text{ g }{\text{g}}^{-1}\right)=\frac{\text{final weight}–\text{initial weight}}{\text{protein intake}},\\ \text{Condition factor}\left(\text{CF},\%\right)=100\times \frac{\text{final weight}\left(\text{g}\right)}{{\left(\text{final length}\left(\text{cm}\right)\right)}^{3}},\\ \text{Hepatosomatic index}\left(\text{HSI},\%\right)=100\times \frac{\text{liver weight}}{\text{body weight}},\\ \text{Gut weight ratio}\left(\%\right)=100\times \frac{\text{gut weight}}{\text{body weight}}. \end{array}$$

## 3. Results

### 3.1. Water Quality and Biofloc Development

**Figure 1:**
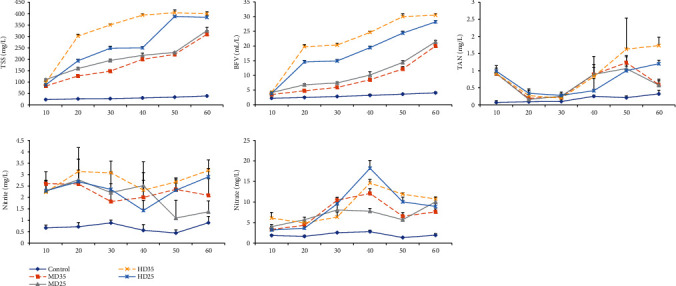
Total suspended solids (TSS), biofloc volume (BFV), total ammonia nitrogen (TAN), nitrite, and nitrate changes in medium (MD) and high (HD) density biofloc tanks and fed 35 and 25% dietary protein and control tanks (clear water, medium density, 35% dietary protein) during the 60-day experiment. Values are means (±SD) of three replications per sampling time in each treatment.

Water quality parameters in biofloc and control tanks during the 60-day trial are shown in Table 2. A significant decrease in pH in the HD biofloc groups was observed. Total dissolved solids (TDS) and electrical conductivity (EC) in the biofloc groups were significantly higher than the control. Total suspended solids (TSS), biofloc volume (BFV), total ammonia nitrogen (TAN), nitrite, and nitrate changes in biofloc and control tanks during the 60-day experiment are presented in Figure 1. The levels of TSS and BFV elevated gradually during the trial. But the BFV in the HD groups increased more quickly than in the other groups. Also, TSS and BFV levels in the HD groups were significantly higher than in the MD groups.

On day 50, MD35 and HD35 biofloc treatments had significantly higher TAN values than the control (clear water) group (Figure 1). On day 30, the nitrite concentration in the HD35 group was significantly higher than in the MD35 and control groups. Also, the lowest nitrite concentration was observed in the MD25 and control groups on day 50. On day 60, higher stocking density led to higher TAN and nitrite. Biofloc groups had significantly higher nitrate values than the control (clear water) group. On days 40 and 50, the nitrate levels in the HD treatments were significantly higher than in the MD treatments.

### 3.2. Proximate Composition of Biofloc

The proximate composition of biofloc in MD and HD biofloc tanks stocked with common carp and fed 35 and 25% dietary protein for 60 days is shown in Table 3. The protein and lipid contents of biofloc were significantly higher in the MD35 group, then the HD35 group, and lower in the MD25 and HD25 groups. The ash content of biofloc was lowest in the MD35 group. The fiber content of biofloc was lowest in the MD35 group and highest in the HD25 group.

### 3.3. Growth Performance and Feed Utilization of Fish

Growth and feed utilization parameters of fish stocked in biofloc systems and control (clear water) for 60 days are presented in Table 4. The final weight, weight gain, and SGR in the MD35 group were significantly higher than in the control and MD25 groups; there were no significant differences between the control and MD25 groups. The final weight, weight gain, and SGR in the HD biofloc groups were significantly lower than in the control. Both stocking density and dietary protein level significantly influenced final weight, weight gain, SGR, FCR, PER, and CF, while interaction between stocking density and dietary protein level did not affect them. The final density was highest in the HD35 group. The highest PER was observed in the MD25 group. The FCR was lower in the MD35 group and higher in the HD25 group compared to the control; there were no significant differences between the control and MD25 groups and between the control and HD35 groups. The condition factor (CF) was lowest in the HD25 group. The hepatosomatic index (HSI) was lowest in HD biofloc treatments.

### 3.4. Digestive and Liver Antioxidant Enzymatic Activities

Digestive enzyme activities in the digestive tract and antioxidant enzyme activities in the liver of fish cultured in biofloc systems and control (clear water) for 60 days are shown in Table 5. The amylase, lipase, and protease activities in the digestive tract of fish cultured in all biofloc groups were significantly higher than in the control (clear water). In the biofloc groups, higher amylase activity was observed in MD groups. The protease activity in MD biofloc groups was significantly higher than in the HD25 group. Also, the lipase activity was lower in the HD25 group.

The liver SOD and GPx activities in fish cultured in all biofloc groups were significantly higher than in the control (clear water). The liver catalase activity in MD35, MD25, and HD35 groups was significantly higher than in the control and HD25 groups. The liver TAOC and MDA levels were significantly affected by stocking density, dietary protein level, and the interaction of these two factors. The liver TAOC level was highest in the MD biofloc groups and lowest in the HD25 group. The liver MDA content was significantly higher in the control and HD25 groups, then the HD35 group, and lower in the MD biofloc groups.

### 3.5. Stress and Innate Immune Parameters

**Figure 2:**
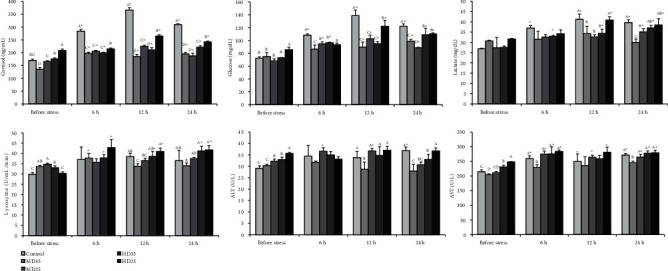
Cortisol, glucose, and lactate levels and lysozyme, ALT, and AST activities of common carp reared in medium (MD) and high (HD) densities and fed 35 and 25% dietary protein in a biofloc system and control (clear water, medium density, 35% dietary protein) for 60 days and after crowding stress (80 kg/m^3^) for 24 h (mean ± SD, *n* = 3). Diverse letters show significant differences (*p* < 0.05) among treatments of each sampling point. Significant differences (*p* < 0.05) between the means obtained before and after crowding stress are marked by asterisks. ALT: alanine aminotransferase; AST: aspartate transaminase.

Cortisol, glucose, and lactate levels and lysozyme, ALT, and AST activities of fish cultured in medium and high densities and fed 35 and 25% dietary protein in a biofloc system and control (clear water, medium density, 35% dietary protein) for 60 days and after acute crowding stress (80 kg/m^3^) for 24 h are presented in Figure 2. Before crowding stress, cortisol and glucose levels in serum were highest in the HD25 group. The lowest serum cortisol value was also observed in the MD35 group. After 12 and 24 h of crowding stress, cortisol and glucose levels in all treatments were significantly higher than the prestress values. The serum cortisol level of common carp was significantly affected by stocking density and the interaction of stocking density and dietary protein level at 24 h after crowding stress (Table 6). At all time points after stress, serum cortisol and glucose values in the biofloc groups were significantly reduced compared to the control (clear water). After 12 h stress, the serum lactate levels in control and HD biofloc treatments were significantly higher than the prestress values. Serum lactate values in MD biofloc treatments were significantly lower at 24 h poststress compared to the control.

Before acute stress, serum lysozyme activity in the MD35, MD25, and HD35 groups was significantly higher than in the control and HD25 groups. After 12 and 24 h stress, lysozyme activity in the HD biofloc groups was significantly higher than those before stress. After 12 h crowding stress, serum lysozyme activity in the MD biofloc groups was significantly lower than in the HD25 group. After 24 h crowding stress, lysozyme activity in the MD35 group was significantly lower than in the HD biofloc groups. The interaction between stocking density and dietary protein level had no effect on serum lysozyme activity of fish at 24 h after crowding stress (Table 6). Before stress, the activities of ALT and AST in serum were highest in the HD25 treatment. After crowding stress, ALT and AST activities were lowest in the MD35 treatment at all sampling times. After 24 h stress, ALT activity in the MD25 group was significantly lower than in the control and HD25 groups.

## 4. Discussion

### 4.1. Water Quality and Biofloc Development

A meaningful decline in pH related to the high abundance of bacteria (higher TSS and BVF levels) in HD biofloc tanks was showed. Bacterial respiration apparently raised the CO_2_ level leading to a corresponding decline in pH [8, 33]. Total suspended solids (TSS) and biofloc volume (BFV) values increased gradually during the trial. But these factors in the HD groups raised more quickly than in the MD tanks because of the higher usage of food, more fish excretion, and nitrogen substance formation [6, 33]. In comparison with the control (clear water), a high level of fluctuation in TAN and nitrite concentrations was observed in the biofloc groups. This fluctuation in the biofloc system is attributed to the change in the bacterial community of biofloc, which includes the heterotrophic assimilation and autotrophic nitrification bacteria [34]. TAN, nitrite, and nitrate levels were little fluctuated in the control group, because the water was exchanged. However, TAN concentration remained at a low level during the trial, not exceeding 1.23 mg L^−1^ (except the HD35 group). On day 60, the lowest TAN levels were observed in the control and MD biofloc groups. Previous studies also showed that TAN concentration was effectively reduced in the BFT groups [8, 35]. Moreover, the lowest nitrite concentrations were observed in the MD25 (1.09 mg L^−1^) and control groups on day 50. On day 60, nitrite concentration was significantly lower in the control and MD25 groups compared to the HD biofloc groups. On day 60, higher stocking density led to higher TAN and nitrite values because of the higher usage of food and more fish excretion. High stocking density combined with the high dietary protein content in HD35 tanks negatively affects the water quality particularly the accumulation of ammonia and nitrite. Although the TAN and nitrite tended to raise in the biofloc groups, the formation and growth of the biofloc in the BFT system were associated with the assimilation of nitrogen substances by heterotrophic bacteria or autotrophic nitrification [6, 36], and therefore, concentrations of TAN and nitrite were controlled. In general, the safe limit of TAN for common carp in the BFT system is less than 1.5 mg L^−1^ [8, 19]. Boyd and Pillai [34] also reported that nitrite levels above 1.0 mg L^−1^ in culture water might cause death to the farmed fish. However, nitrite quickly converts to the more stable form (nitrate) in biofloc environment. Biofloc groups had significantly higher nitrate values than the control (clear water), because the nitrifiers were active in the biofloc tanks ([23]). Therefore, heterotrophic and nitrifier bacteria formed in biofloc tanks can maintain the ammonia and nitrite at safe levels for common carp when reared in MD.

### 4.2. Proximate Composition of Biofloc

In common carp juvenile diets, 35–40% crude protein is the normal selection [19]. The results of our experiment indicated that crude protein content of biofloc was significantly higher in the MD35 group (34.2%), then the HD35 group (29.2%), and lower in the MD25 (22.3%) and HD25 (21.8%) groups. Similarly, the fish fed higher dietary protein had significantly higher crude lipid content than those fed lower dietary protein. The ash and fiber contents of biofloc were lowest in the MD35 group. So, the biofloc quality in the MD35 biofloc group was more appropriate (in terms of nutrition) than in the other groups. The microbial flocs quality in terms of fish nutrition in our experiment was suitable for common carp except for the low crude lipid contents. Azim and Little [23] reported 37.9–38.4% crude protein, 3.1–3.2% crude lipid, 11.8–13.3% ash, 5.7-6.2% fiber, and 18.6–19 kJ/g energy in the biofloc obtained from indoor tanks stocked with Nile tilapia and fed diets containing 35 or 24% crude protein. However, a higher quality biofloc was estimated in a similar system without fish exhibiting that *in situ* biofloc utilization by fish might have an impact on the proximate composition of biofloc ([37]).

### 4.3. Growth Performance and Feed Utilization of Fish

In our study, the final weight, weight gain, and SGR in the HD biofloc groups were significantly lower than in the control, while FCR was significantly increased in the HD25 group compared to the control, indicating an inefficiency of biofloc for common carp growth in high density. Corroborating to our study, previous studies reported that the increase in stocking density tends to decrease the growth of fish in biofloc systems [9, 38]. At high density, the higher feed supply causes lower water quality and increases competition for space and feed, resulting in decreased growth [10]. This situation increases risks of stress to the fish that impairs their physiology and immune system [38]. The raised energy requirement associated with high density stress has an adverse effect on FCR ([39]). Also, the HSI was lowest in HD treatments. An elevated mobilization of liver stores due to greater energy requirement in high density situation may be responsible for less HSI in the HD groups [8, 40].

Overall, the final weight, weight gain, and SGR in the MD35 biofloc group were significantly higher than the control and other biofloc treatments. Also, the FCR was lower in the MD35 group compared to the control and HD biofloc groups. This indicates that MD biofloc systems could be used to improve the growth performance of common carp. The favorable effects of the biofloc system on fish production might be due to different reasons, the biofloc system provides better and more stable water quality [4, 8], microbial flocs served as a supplemental food source for the fish [10], and biofloc consumption enhanced the fish resistance against stress [9, 10]. Biofloc is a good nutritional source of proteins, lipids, vitamins, minerals, phosphorus, and probiotics for cultured fish [5, 7, 23]. Furthermore, biofloc has some bioactive compounds such as carotenoids, phytosteroids, exogenous microbial enzymes, and endogenous digestive enzymes which may promote the digestion of food resulting in enhanced food absorption [8, 9, 20].

In this study, the FCR was lower in the MD35 biofloc group compared to the control; there were no significant differences between the MD35 and MD25 biofloc groups. Also, the highest PER was observed in the MD25 biofloc group indicating that the reduction of food protein in the biofloc system can improve protein utilization and spare food protein [3]. Ebrahimi et al. [19] reported that biofloc could not compensate for 15% protein deficiency (drop from 35% to 20%) in common carp juvenile diet, but our study indicated that biofloc could compensate 10% reduction of protein (drop from 35% to 25%) in common carp juvenile diet. The enhancement in growth performance and feed utilization in our study may be a result of the activity of heterotrophic bacteria, which could assimilate the nitrogen substances and supply microbial protein for fish consumption [3, 4]. Therefore, the lower protein requirement of fish reared in the biofloc system could be explained by the fish consuming biofloc, which has high nutritional importance, particularly in terms of protein.

### 4.4. Digestive and Liver Antioxidant Enzymatic Activities

The amylase, lipase, and protease activities in common carp cultured in the biofloc groups were significantly higher than in the control (clear water). In both MD and HD biofloc groups, higher lipase activity was observed in 35% dietary protein groups. Similarly, Khorasaninasab, Keyvanshokooh, Pasha-Zanoosi, and Shahriari [41] exhibited an increase in pepsin (stomach) and trypsin (intestine) activities of Nile tilapia in the biofloc system as dietary protein levels increased. Biofloc presented relatively high protease and amylase activities ([42]). These microbial enzymes can improve the breakdown of nutritional ingredients in the food and probably promote food digestion and absorption [43]. Also, biofloc has a stimulatory impact on the activities of digestive enzymes, which may facilitate the utilization of food and enhance the growth of fish [8, 44]. In our study, in the biofloc groups, higher amylase activity was observed in the MD groups. The protease activity in the MD groups was significantly higher than in the HD25 group. The lipase activity was lower in the HD25 group. Adineh et al. [8] also reported that the amylase activity reduced significantly with elevating culture density. So, higher culture density may damage the digestion and utilization of the food in the fish.

The stress response may raise free radicals, resulting in higher lipid peroxidation levels and further oxidative damage [45]. Antioxidant enzymes can prevent lipid peroxidation in different tissues. In the present study, the activities of SOD and GPx in the liver of fish cultured in all biofloc treatments were significantly higher than the control, suggesting that biofloc can improve antioxidant status in common carp. The liver catalase activity in the MD35, MD25, and HD35 groups was significantly higher than in the control and HD25 groups. The liver TAOC level was highest and MDA content was lowest in the MD biofloc groups. In the study of Adineh et al. [8], the MDA levels in the common carp reared in the biofloc tanks at 6 and 12 kg/m^3^ stocking densities were significantly lower than the fish cultured in the clear water tanks at 12 kg/m^3^ stocking density. Also, elevated SOD and catalase activities were reported in Nile tilapia cultured in biofloc conditions compared with fish cultured in clear water [14]. In the HD25 group, the fish could tolerate little stocking density stress than in the other biofloc groups. So, stress could decrease the antioxidant capacity of fish under high density conditions; even the biofloc *in situ* had anticrowding stress efficacy [8, 9]. In agreement with our findings, the SOD activity and glutathione content of Nile tilapia in the control (clear water) and high density biofloc groups were significantly lower than those in the low and middle density biofloc groups [9]. In the HD biofloc groups, a significant increase in GPx and catalase activities and TAOC level and lower MDA value observed in high dietary protein level (35%) might reflect increased fish health and reduced oxidative stress. In agreement with our results, the common carp juveniles fed 30% dietary protein and reared in the BFT groups showed better antioxidant status than those fed 20% dietary protein and reared in control [19].

### 4.5. Stress and Innate Immune Responses

Stress that induces elevated blood cortisol levels can also suppress immune responses of fish [46]. Before acute crowding stress, the serum cortisol level was lower in the MD35 group and higher in the HD25 group compared to the control (clear water). The serum glucose level was also highest in the HD25 group. Our results exhibit that when the dietary protein content in the MD biofloc groups reduced from 35% to 25%, the stress resistance of common carp decreased, but no remarkable difference was observed when compared to the control. Also, serum lysozyme activity in the MD35, MD25, and HD35 groups was significantly higher than in the control and HD25 groups. Therefore, biofloc can be used as a protein source and immune strengthener in common carp. Similarly, Yu et al. [16] indicated that despite the low protein level in diet, the biofloc system has the potential to mitigate copper-induced immunosuppression and oxidative stress in juvenile *R. lagowski*. Moreover, lower levels of cortisol and glucose and higher lysozyme activity were reported in biofloc tanks at stocking densities up to 250 fish/m^3^ compared to the control (clear water, 150 fish/m^3^) [38]. Higher lysozyme activities in biofloc may be due to the natural probiotic effect [38]. According to Liu et al. [9], lysozyme values were significantly lower in fish cultured in the control and high density biofloc treatments as compared to the low and middle density biofloc treatments. In the present experiment, the immune function was impaired in the control fish (similar to the HD25 treatment), though the stocking density was lower and dietary protein level was higher than the HD25 treatment, possibly because there was no biofloc in the control group. Microbial flocs continuously provide additional protein, polyunsaturated fatty acids, bioactive products, carotenoids, vitamins, minerals, and some probiotic microorganisms [5, 9, 23, 47, 48]. Microbial flocs may act as an immunostimulant [14, 49]. The complementary protein source rich in essential amino acids provided by the biofloc system might also contribute to the immune function of fish [50].

After 12 and 24 h of crowding stress, serum cortisol and glucose levels in all treatments were significantly higher than the prestress values. At all time points after acute stress, cortisol and glucose values in the biofloc treatments were significantly decreased compared with the control (clear water). These results indicated that biofloc could alleviate the elevation in cortisol and glucose caused by acute stress. This was confirmed by the results of Fauji et al. [10], showing that the African catfish reared in biofloc conditions had higher resistance to high salinity stress and bacterial challenge. The results of the present study demonstrated that the biofloc *in situ* had anticrowding stress efficacy. We concluded that common carp reared in biofloc conditions could raise the ability to resist stress. Moreover, our findings exhibited that in the HD biofloc groups, 35% dietary protein (HD35) is more efficient than 25% dietary protein (HD25) in preventing the increase in cortisol levels induced by crowding stress.

In the present study, increased stocking density influenced the acute crowding stress response in common carp. After 24 h crowding stress, cortisol and glucose values in HD biofloc groups were significantly higher than in the MD biofloc groups, and serum lactate levels in the HD biofloc groups were significantly higher compared to the MD35 biofloc group. These results reveal that common carp reared in HD biofloc tanks were more susceptible to acute crowding stress compared to the MD biofloc groups. In agreement with our findings, after 15 h crowding, plasma cortisol concentrations of common carp in crowded plus confinement treatments were higher than in the control and confinement treatments [12]. Serum lactate levels in the MD biofloc groups were significantly lower at 24 h poststress compared to the control. After the occurrence of acute stress, lactate levels of the MD35 group were also unchanged over the course of the test. These findings indicated that the MD biofloc system can exert beneficial impacts on the welfare of acutely stressed common carp.

After 12 and 24 h stress, lysozyme activity in HD treatments was significantly higher than before stress. The fish in HD treatments may have enhanced lysozyme activities to further stress resistance [51]. Similar findings have been showed in the common carp [8, 52]. However, after 12 h crowding stress, lysozyme activity in the MD35 biofloc group was significantly lower than in the control and HD biofloc groups. After 24 h crowding stress, lysozyme activity in the MD35 group was significantly lower than in the HD biofloc groups. These findings indicated that the MD biofloc system with 35% dietary protein can mitigate the elevation in lysozyme activity caused by acute stressors.

The activities of ALT and AST in serum can be used as important indicators of the function of the fish liver, as the increased permeability of damaged hepatocytes causes a release of these enzymes into the blood [53]. These enzymes also can be used as an indicator of stress in fish [38]. Before the application of acute stress, the activities of ALT and AST in serum were highest in the common carp reared in high density and fed 25% dietary protein (HD25 group). Also, ALT and AST values raised with the increase in stocking density revealed that liver health was affected by the high density stress. Similar to our results, increased levels of ALT and AST were reported in the serum of fish with increasing stocking density [8, 9, 38]. After the occurrence of crowding stress, the activities of ALT and AST were lowest in the MD35 group at all sampling times. Furthermore, after 24 h crowding stress, ALT activity in the MD25 group was significantly lower than in the control and HD25 groups. Our results indicate that the MD biofloc system can reduce liver damage in fish subjected to acute stress.

In conclusion, our study demonstrated that the growth of juvenile common carp in the HD biofloc groups was significantly lower than in the control, while FCR was increased in the HD25 biofloc group compared to the control, suggesting an inefficiency of biofloc for fish growth in HD. Our results revealed that the biofloc system with medium stocking density could reduce oxidative stress and improve growth performance, feed utilization, digestive activity, immune status, health, and resistance to additional acute stress in fish. Furthermore, high dietary protein levels could improve growth, lipase activity, antioxidant status, and stress response. Therefore, the culture of juvenile common carp in the biofloc system at an initial stocking density of 10 kg/m^3^ could be recommended. Also, microbial flocs as a protein source could compensate 10% reduction of protein in common carp juvenile diet when reared in MD.

## Figures and Tables

**Table 1:** Formulation and chemical composition (% dry matter basis) of the experimental diets containing two levels of crude protein (CP) for common carp.

Ingredients (g/kg diet)	Experimental diets	
	35% CP	25% CP
Fish meal (72.72% CP)	100	70
Meat meal (poultry, 58.5% CP)	200	130
Soybean meal (46.78% CP)	230	120
Wheat flour (14.52% CP)	349	270
Corn flour (7.77% CP)	90	379
Fish oil	7	7
Soybean oil	7	7
Lysine	7	7
Methionine	5	5
Vitamin premix^a^	2.5	2.5
Mineral premix^b^	2.5	2.5
Total	1000	1000
*Chemical composition (%)*		
Dry matter	86.08	86.23
Crude protein	35.12	24.89
Crude lipid	5.10	5.63
Crude fiber	2.34	2.55
Ash	4.20	5.78

^a^Vitamin premix (per kg of diet): A: 1000 IU; D3: 5000 IU; E: 20 mg; B5: 100 mg; B2: 20 mg; B6: 20 mg; B1: 20 mg; H: 1 mg; B9: 6 mg; B12: 1 mg; B4: 600 mg; C: 50 mg. ^b^Mineral premix (per kg of diet): Mg: 350 mg; Fe: 13 mg; Co: 2.5 mg; Cu: 3 mg; Zn: 60 mg; NaCl: 3 g; dicalcium phosphate: 10 g.

**Table 2:** The means of water quality parameters in medium (MD) and high (HD) density biofloc tanks stocked with common carp and fed 35 and 25% dietary protein and control tanks (clear water, medium density, 35% dietary protein) for 60 days (mean ± SD, *n* = 180).

Parameters	Experimental groups				
	Control	MD35	MD25	HD35	HD25
Temperature (°C)	23.53 ± 0.81	23.64 ± 0.55	23.44 ± 0.77	23.72 ± 0.44	23.66 ± 0.33
Salinity (g L^−1^)	0.54 ± 0.01	0.55 ± 0.07	0.53 ± 0.06	0.57 ± 0.09	0.54 ± 0.07
DO (mg L^−1^)	7.42 ± 0.14	7.12 ± 0.29	7.26 ± 0.28	7.17 ± 0.39	7.09 ± 0.52
pH	7.84 ± 0.15^ab^	7.86 ± 0.09^a^	7.80 ± 0.10^ab^	7.67 ± 0.15^c^	7.71 ± 0.13^bc^
TDS (mg L^−1^)	440.33 ± 4.15^b^	558.22 ± 71.93^a^	536.33 ± 46.67^a^	579.33 ± 90.91^a^	560.44 ± 68.81^a^
EC (*μ*s cm^−1^)	897.77 ± 8.33^b^	1128.44 ± 141.43^a^	1086.77 ± 98.00^a^	1154.44 ± 167.84^a^	1134.22 ± 135.47^a^

Means in the same row with different superscripts are significantly different (*p* < 0.05). DO: dissolved oxygen; TDS: total dissolved solids; EC: electrical conductivity.

**Table 3:** Proximate composition of biofloc in medium (MD) and high (HD) density biofloc tanks stocked with common carp and fed 35 and 25% dietary protein for 60 days (mean ± SD, *n* = 3).

Parameters	Experimental groups				Two-way ANOVA		
	MD35	MD25	HD35	HD25	Density	Protein	Interaction
Dry matter (%)	89.48 ± 0.80	89.26 ± 0.96	88.34 ± 1.17	88.12 ± 0.98	NS	NS	NS
Crude protein (%DM)	34.21 ± 0.44^a^	22.30 ± 0.20^c^	29.24 ± 0.51^b^	21.86 ± 0.23^c^	*p* < 0.001	*p* < 0.001	*p* < 0.001
Crude lipid (%DM)	1.40 ± 0.01^a^	0.78 ± 0.01^c^	1.01 ± 0.03^b^	0.80 ± 0.01^c^	*p* < 0.001	*p* < 0.001	*p* < 0.001
Ash (%DM)	3.05 ± 0.05^c^	3.60 ± 0.10^a^	3.34 ± 0.08^b^	3.50 ± 0.11^ab^	NS	*p* < 0.001	*p* = 0.006
Crude fiber (%DM)	0.80 ± 0.01^c^	0.92 ± 0.01^b^	0.90 ± 0.02^b^	0.98 ± 0.03^a^	*p* < 0.001	*p* < 0.001	NS

Means in the same row with different superscripts are significantly different (*p* < 0.05). NS: not significant (*p* < 0.05).

**Table 4:** Growth performance and feed utilization of common carp reared in medium (MD) and high (HD) densities and fed 35 and 25% dietary protein in a biofloc system and control (clear water, medium density, 35% dietary protein) for 60 days (mean ± SD, *n* = 3).

Parameters	Experimental groups					Two-way ANOVA		
	Control	MD35	MD25	HD35	HD25	Density	Protein	Interaction
Initial weight (g)	12.03 ± 1.06	12.42 ± 0.92	12.34 ± 1.01	12.01 ± 0.95	11.94 ± 0.99	*p* = 0.009	NS	NS
Final weight (g)	26.71 ± 1.86^b^	29.21 ± 2.28^a^	27.47 ± 1.82^b^	22.90 ± 2.01^c^	20.39 ± 1.85^d^	*p* < 0.001	*p* < 0.001	NS
Initial density (kg m^−3^)	10.02 ± 0.88^b^	10.35 ± 0.77^b^	10.29 ± 0.84^b^	20.01 ± 1.59^a^	19.90 ± 1.65^a^	*p* < 0.001	NS	NS
Final density (kg m^−3^)	22.26 ± 1.55^d^	24.34 ± 1.90^c^	22.89 ± 1.51^d^	38.17 ± 3.36^a^	34.00 ± 3.09^b^	*p* < 0.001	*p* < 0.001	*p* = 0.003
Survival (%)	100	100	100	100	100	NS	NS	NS
Weight gain (%)	123.98 ± 27.29^b^	136.07 ± 20.80^a^	123.95 ± 23.46^b^	91.95 ± 22.44^c^	71.59 ± 18.05^d^	*p* < 0.001	*p* < 0.001	NS
SGR (% day^−1^)	1.33 ± 0.19^b^	1.42 ± 0.14^a^	1.33 ± 0.17^b^	1.07 ± 0.19^c^	0.89 ± 0.17^d^	*p* < 0.001	*p* < 0.001	NS
FCR (g g^−1^)	1.77 ± 0.31^bc^	1.54 ± 0.19^d^	1.71 ± 0.27^cd^	1.93 ± 0.52^b^	2.24 ± 0.63^a^	*p* < 0.001	*p* = 0.003	NS
PER (g g^−1^)	1.65 ± 0.29^c^	1.88 ± 0.24^b^	2.38 ± 0.34^a^	1.56 ± 0.37^c^	1.91 ± 0.50^b^	*p* < 0.001	*p* < 0.001	NS
CF (%)	1.55 ± 0.13^ab^	1.69 ± 0.22^a^	1.58 ± 0.15^ab^	1.49 ± 0.22^b^	1.32 ± 0.16^c^	*p* = 0.001	*p* = 0.035	NS
HSI (%)	1.59 ± 0.40^a^	1.98 ± 0.13^a^	1.74 ± 0.37^a^	0.84 ± 0.18^b^	0.93 ± 0.11^b^	*p* < 0.001	NS	NS
Gut weight ratio (%)	9.22 ± 0.23	9.67 ± 0.33	8.85 ± 1.01	8.46 ± 1.25	8.21 ± 1.12	NS	NS	NS

Means in the same row with different superscripts are significantly different (*p* < 0.05). NS: not significant (*p* > 0.05). Weight gain (%) = 100 × (final weight–initial weight)/initial weight. Specific growth rate (SGR, %day^−1^) = 100 × (ln final weight–ln initial weight)/days. Feed conversion ratio (FCR, g g^−1^) = dry feed intake/(final weight–initial weight).Protein efficiency ratio (PER, g g^−1^) = (final weight–initial weight)/protein intake.Condition factor (CF, %) = 100 × (final weight (g)/(final length (cm))^3^).Hepatosomatic index (HSI, %) = 100 × (liver weight/body weight).Gut weight ratio (%) = 100 × (gut weight/body weight).

**Table 5:** Digestive enzyme activities in the digestive tract and antioxidant enzyme activities in the liver of common carp reared in medium (MD) and high (HD) densities and fed 35 and 25% dietary protein in a biofloc system and control (clear water, medium density, 35% dietary protein) for 60 days (mean ± SD, *n* = 3).

Parameters	Experimental groups					Two-way ANOVA		
	Control	MD35	MD25	HD35	HD25	Density	Protein	Interaction
Amylase (U mg^−1^ protein)	8.90 ± 0.21^c^	11.53 ± 0.17^ab^	12.25 ± 0.63^a^	11.25 ± 0.40^b^	11.14 ± 0.51^b^	*p* = 0.032	NS	NS
Lipase (U mg^−1^ protein)	0.76 ± 0.05^d^	1.48 ± 0.04^a^	1.33 ± 0.14^b^	1.31 ± 0.03^b^	1.09 ± 0.03^c^	*p* = 0.002	*p* = 0.004	NS
Protease (U mg^−1^ protein)	2.33 ± 0.25^c^	3.36 ± 0.04^a^	3.38 ± 0.01^a^	3.25 ± 0.12^ab^	3.11 ± 0.02^b^	*p* = 0.001	NS	NS
SOD (U mg^−1^ protein)	2.03 ± 0.07^c^	2.90 ± 0.13^a^	2.72 ± 0.02^ab^	2.56 ± 0.18^b^	2.61 ± 0.05^b^	*p* = 0.010	NS	NS
GPx (U mg^−1^ protein)	4.22 ± 0.34^c^	5.83 ± 0.49^a^	5.36 ± 0.35^ab^	5.91 ± 0.12^a^	4.97 ± 0.05^b^	NS	*p* = 0.004	NS
Catalase (U mg^−1^ protein)	1.40 ± 0.01^b^	1.69 ± 0.07^a^	1.59 ± 0.03^a^	1.68 ± 0.07^a^	1.46 ± 0.06^b^	NS	*p* = 0.003	NS
MDA (nmol mg^−1^ protein)	1.68 ± 0.03^a^	1.31 ± 0.04^c^	1.27 ± 0.04^c^	1.47 ± 0.02^b^	1.70 ± 0.01^a^	*p* < 0.001	*p* = 0.001	*p* < 0.001
TAOC (nmol mg^−1^ protein)	21.51 ± 0.10^b^	27.59 ± 1.00^a^	27.15 ± 0.95^a^	21.99 ± 0.30^b^	17.80 ± 0.70^c^	*p* < 0.001	*p* = 0.001	*p* = 0.003

Means in the same row with different superscripts are significantly different (*p* < 0.05). SOD: superoxide dismutase; GPx: glutathione peroxidase; MDA: malondialdehyde; TAOC: total antioxidant capacity; NS: not significant (*p* < 0.05).

**Table 6:** Two-way ANOVA showing the effects of stocking density, dietary protein level, and their interactions on stress and innate immune responses of common carp in a biofloc system before (at the end of 60-day experiment) and after crowding stress (80 kg/m^3^) for 24 h.

Parameters		Two-way ANOVA		
		Density	Protein	Interaction
Cortisol	Before stress	*p* < 0.001	*p* < 0.001	NS
	6 h	*p* = 0.017	*p* < 0.001	NS
	12 h	*p* < 0.001	*p* < 0.001	NS
	24 h	*p* < 0.001	NS	*p* = 0.007

Glucose	Before stress	*p* = 0.009	NS	*p* = 0.003
	6 h	NS	NS	*p* = 0.031
	12 h	*p* = 0.013	*p* = 0.001	NS
	24 h	*p* = 0.001	NS	NS

Lactate	Before stress	NS	NS	*p* = 0.033
	6 h	NS	NS	NS
	12 h	*p* = 0.012	NS	*p* = 0.014
	24 h	*p* = 0.002	*p* = 0.023	NS

Lysozyme	Before stress	*p* < 0.001	NS	*p* = 0.002
	6 h	*p* = 0.037	NS	*p* = 0.043
	12 h	*p* = 0.001	*p* = 0.029	NS
	24 h	*p* < 0.001	NS	NS

ALT	Before stress	*p* < 0.001	*p* = 0.001	NS
	6 h	NS	NS	*p* = 0.001
	12 h	NS	*p* = 0.011	NS
	24 h	*p* = 0.001	*p* = 0.024	NS

AST	Before stress	*p* < 0.001	*p* = 0.001	*p* = 0.041
	6 h	*p* = 0.009	*p* = 0.010	NS
	12 h	NS	*p* = 0.046	NS
	24 h	*p* = 0.001	NS	NS

ALT: alanine aminotransferase; AST: aspartate transaminase; NS: not significant (*p* < 0.05).

## Data Availability

The data are available from the corresponding author upon reasonable request.
